# Supplementary figures and images for: Relationship between Fatigue and Self-Perception of Constipation in Community-Dwelling Older Adults during the COVID-19 Pandemic
Source: Int J Environ Res Public Health. 2022 Jul 9;19(14):8406. doi: 10.3390/ijerph19148406 (PMC9323158; doi:10.3390/ijerph19148406)

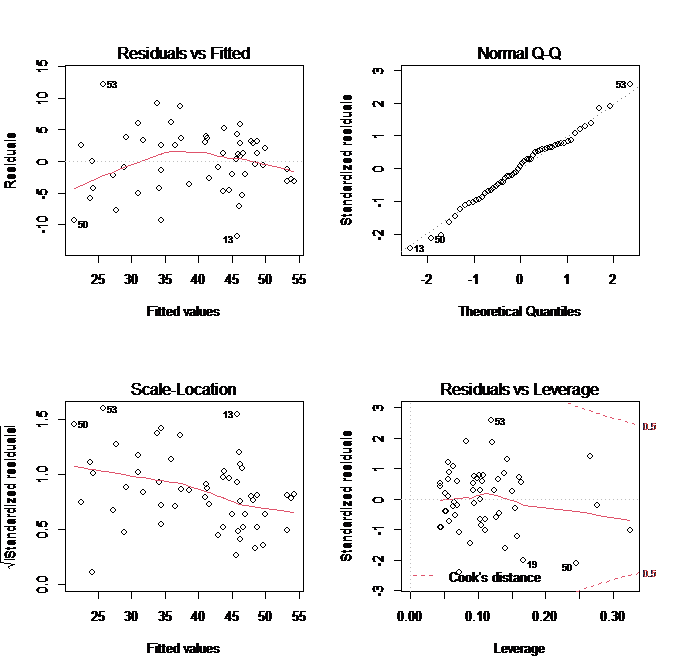

Supplement: Supplementary file 1 [file ijerph-19-08406-s001.zip › ijerph-1764028-supplementary.tif]
